# A streamlined protocol for small-scale protoplast generation and CRISPR/Cpf1-mediated genome editing in *Fusarium oxysporum*

**DOI:** 10.1016/j.isci.2026.117365

**Published:** 2026-08-27

**Authors:** Jun-Ze Zheng, Szu-Chieh Huang, Wen-Ting Zeng, Ying-Hong Lin, Tao-Ho Chang

**Affiliations:** 1 Program in Plant Health Care, Academy of Circular Economy, National Chung Hsing University, Nantou 540, Taiwan; 2 Department of Plant Medicine, National Pingtung University of Science and Technology, Pingtung 912, Taiwan

**Keywords:** *Fusarium oxysporum*, gene editing, CRISPR/Cpf1, protoplast, ribonucleoprotein transformation, plant-microbe interaction

## Abstract

*Fusarium oxysporum* is a significant threat to agriculture and One Health, requiring advanced molecular tools for functional genomic analyses and biological control agent development. Existing gene-editing methods are hampered by costly protoplast preparation protocols and by CRISPR-Cas9 limitations, such as restricted protospacer adjacent motif (PAM) sequences and complex guide RNA requirements. We engineered an efficient CRISPR/Cpf1 system that overcomes these issues through three main innovations: small-scale protoplast generation using filter column-based methods that greatly reduce enzyme consumption while simplifying workflows, a CRISPR/Cpf1 system with shorter guide RNA design and staggered DNA cleavage to promote homologous recombination, and minimal homology arm strategies that significantly decrease cloning complexity. Extensive validation confirms successful gene targeting with molecular verification and functional analysis via standardized pathogenicity assays. This integrated platform offers affordable, accessible tools for systematic *F. oxysporum* research, enhancing fundamental understanding of plant-pathogen interactions and supporting high-throughput screening vital for agricultural biotechnology and biological agent development.

## Highlights

• Reduced enzyme costs by 95% through small-scale protoplast preparation
• CRISPR/Cpf1 system established for efficient gene editing in *Fusarium oxysporum*
• Streamlined workflow enables routine gene targeting and rapid mutant screening
• Complete workflow validated with EGFP-marked pathogenicity

## Graphical abstract

**Figure:**
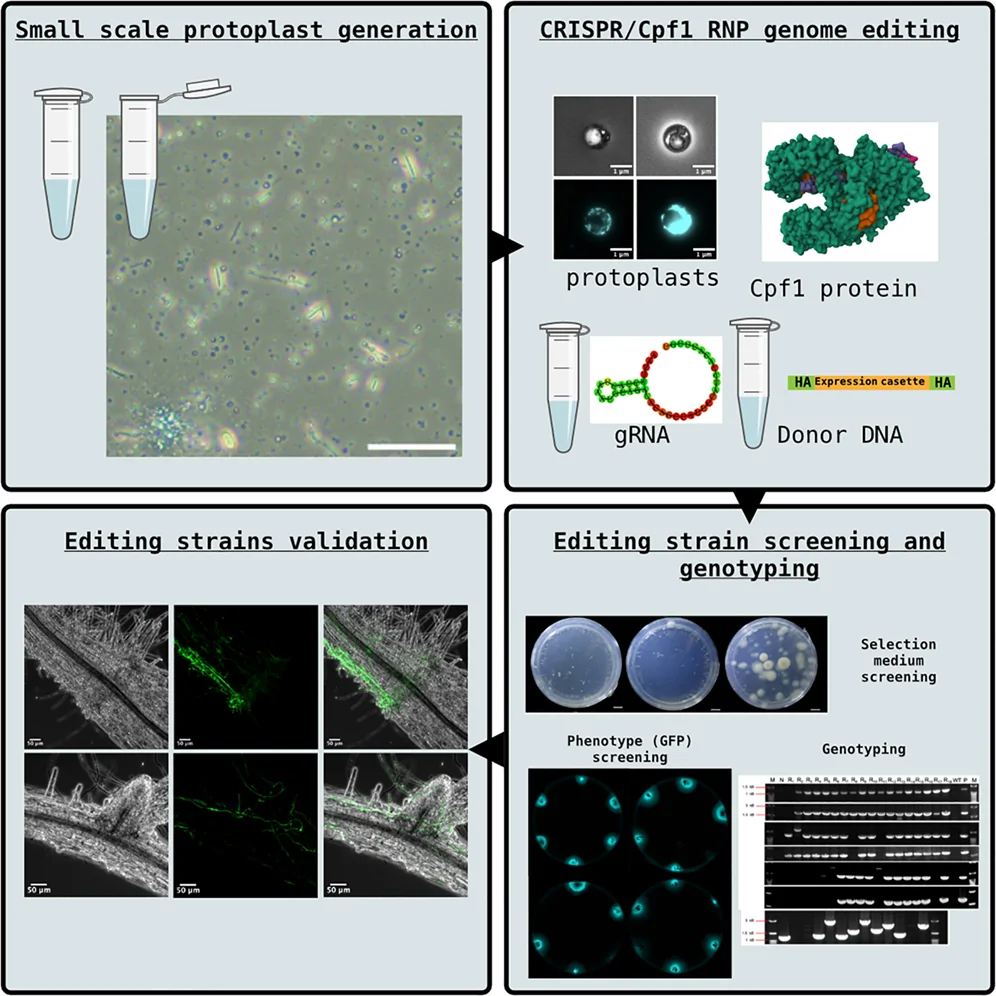

## Teaser

Biotechnology; Plant biotechnology; Plant biology; Interaction of plants with organisms; Molecular plant pathology

## Introduction

*Fusarium oxysporum* represents one of the most economically important soilborne plant pathogens worldwide, causing vascular wilt diseases that result in substantial annual crop losses.^1^^,^^2^ This highly adaptable pathogen exhibits remarkable host specificity through specialized forms (*formae speciales*) that have co-evolved with specific plant families, making it an ideal model system for understanding plant-pathogen interactions.^3^ Advancing knowledge of *F. oxysporum* virulence mechanisms and developing effective disease management strategies require robust molecular tools for systematic gene function analysis, which are essential for both fundamental research and practical applications in crop protection and biotechnology.

Despite the crucial role of molecular research in *F. oxysporum*, progress in functional genomics faces major challenges due to technical barriers. Fungal transformation remains difficult, involving labor-intensive protoplast preparation with costly enzymatic mixes and precise execution.^4^ Conventional methods demand large amounts of cell wall-degrading enzymes, making large-scale experiments cost-prohibitive.^5^ Additionally, these workflows are time consuming, often taking extended periods from protoplast creation to mutant confirmation. These challenges restrict the scope of systematic studies of gene function and slow progress in understanding the molecular basis of pathogenicity and host specificity.

Current CRISPR-Cas9 technology has significantly advanced fungal molecular biology, providing enhanced precision for targeted gene disruption and modification across diverse fungal species.^6^ CRISPR-Cas9 systems have been successfully implemented in several *Fusarium* species, enabling targeted gene knockouts and facilitating investigations of virulence mechanisms.^7^^,^^8^^,^^9^ However, traditional CRISPR-Cas9 approaches in fungi require extensive homology arms (typically 500–2,000 bp) for reliable homology-directed repair.^10^^,^^11^ The blunt-end DNA cuts generated by Cas9 can activate non-homologous end joining (NHEJ) repair pathways, which are error-prone and often result in imprecise gene modifications rather than targeted homologous recombination.^12^ These characteristics necessitate complex experimental designs and may limit the efficiency of precise gene-targeting applications in fungal systems.

The emergence of CRISPR/Cpf1 (Cas12a) technology offers distinct advantages that may address these limitations.^13^^,^^14^^,^^15^ Cpf1 generates staggered DNA breaks with 4–5 nucleotide overhangs that promote homologous recombination and reduce reliance on error-prone NHEJ pathways. Additionally, Cpf1 requires a 5′-TTTV protospacer adjacent motif (PAM), which broadens target-site accessibility, making it particularly suitable for editing AT-rich genomic regions.^15^ The system also utilizes shorter guide RNAs that require no complex scaffold, potentially simplifying experimental protocols.^15^ Despite these theoretical advantages, the application of the CRISPR/Cpf1 system has not yet been established in *F. oxysporum*.

This study aims to develop and validate an optimized CRISPR/Cpf1 system for *F. oxysporum* transformation that addresses current technical and economic limitations. Our objectives were to establish streamlined protoplast preparation protocols, systematically evaluate Cpf1-mediated gene editing capabilities, and integrate these innovations into a comprehensive workflow suitable for high-throughput functional genomics applications.

## Results

### Filter column-based design for small-scale protoplast isolation

**Scheme 1:**
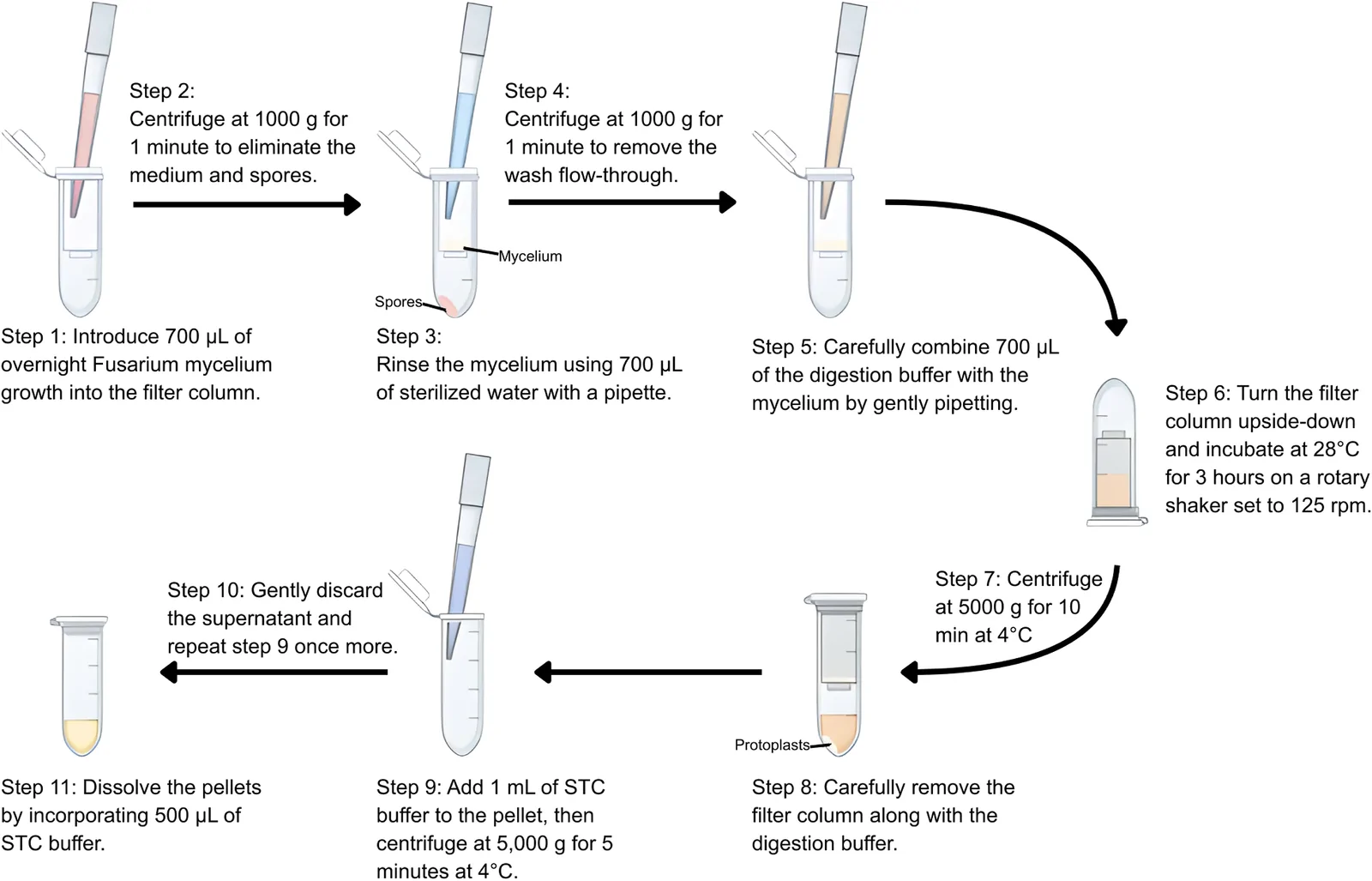
Filter column-based design for small-scale protoplast isolation Step-by-step workflow showing mycelium loading into filter columns, washing procedures, enzymatic digestion in inverted columns with rotary shaking, centrifugation-based protoplast recovery, and final resuspension in SuTC buffer.

This filter column-based approach streamlines the entire protoplast preparation workflow and significantly reduces enzyme consumption, addressing the complexity and cost barriers of traditional methods (Scheme 1). The design employs a disposable filter column as both reaction vessel and separation device, allowing gentle processing of fungal mycelium through controlled washing, digestion, and collection steps. The protocol begins with loading overnight-grown *Fusarium* mycelium directly into the filter column, followed by sequential washing to remove growth medium and spores. Enzymatic digestion occurs within the inverted column under gentle rotary shaking, and protoplasts are recovered through controlled centrifugation steps that minimize mechanical stress while maximizing yield.

**Figure 1:**
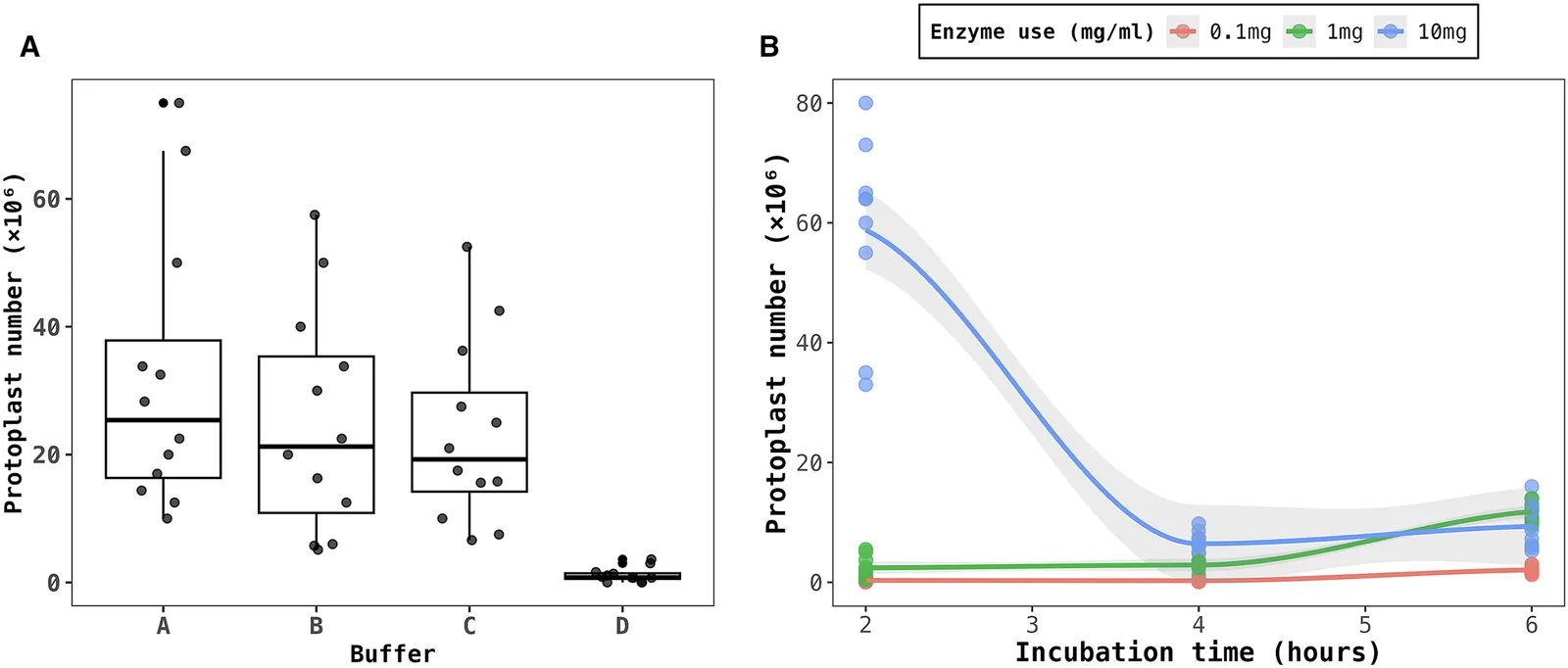
Protoplast isolation experiments (A) Protoplast yields using different buffer systems: A, 0.8 M NaCl; B, 0.8 M KCl; C, 10 mM Na_2_HPO_4_, 20 mM CaCl_2_, and 1.2 M NaCl, pH 5.8; and D, 0.6 M mannitol, 10 mM Tris-HCl, and 10 mM CaCl_2_, pH 7.5. Within each boxplot, the horizontal center line represents the median, the box limits denote the interquartile range (IQR, 25th to 75th percentiles), and the whiskers extend to 1.5 × IQR. Individual dots represent independent data points. (B) Time-course analysis of protoplast production using different Driselase concentrations (0.1, 1, and 10 mg per reaction) over 2–6 h incubation. Individual measurements are plotted as points, with solid lines representing locally estimated scatterplot smoothing (LOESS) regression curves and shaded areas indicating 95% confidence intervals.

Protoplast isolation optimization revealed significant performance differences across experimental conditions (Figures 1 and S1). Buffer system comparison demonstrated that both 0.8 M NaCl and 0.8 M KCl solutions yielded comparable protoplast densities (median ∼25–30 × 10^6^ cells/mL), significantly outperforming phosphate-based and mannitol-based buffers, which achieved less than 5 × 10^5^ cells/mL (Figure 1A). Systematic enzyme concentration analysis showed that the filter column approach achieved optimal protoplast production (highest yield of around 8.0 × 10^7^ cells/mL at 2 h) with 7 mg Driselase and 10 mg β-glucanase per 0.7 mL reaction (Figure 1B).

**Figure 2:**
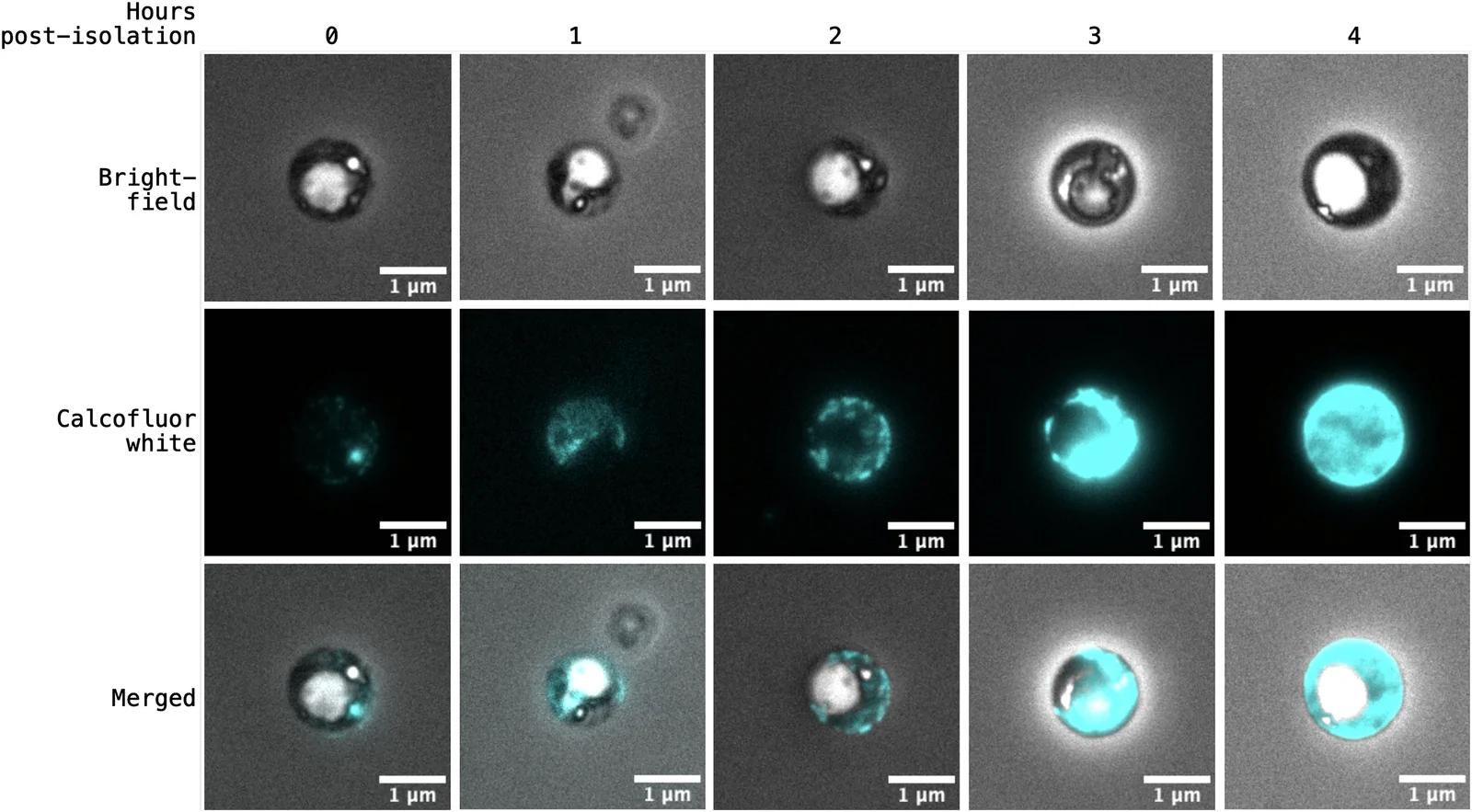
Time-course analysis of cell wall regeneration Representative protoplasts showing cell wall reformation at 0, 1, 2, 3, and 4 h post-isolation. Images captured under bright-field illumination (top row), calcofluor white fluorescence staining (middle row), and merged channels (bottom row). Calcofluor white specifically stains cellulose and chitin components of fungal cell walls. Scale bars represent 1 μm.

Time-course analysis of cell wall regeneration showed quick rebuilding patterns essential for transformation timing (Figure 2). Calcofluor white staining indicated that freshly isolated protoplasts had minimal cell wall material at 0 h post-isolation, with gradual wall rebuilding visible by 1–2 h and complete reformation by 4 h. This swift regeneration process requires immediate transformation after protoplast preparation to achieve maximum efficiency.

### Systematic design and *in vitro* validation of URA5-targeting gRNAs for CRISPR/Cpf1

**Figure 3:**
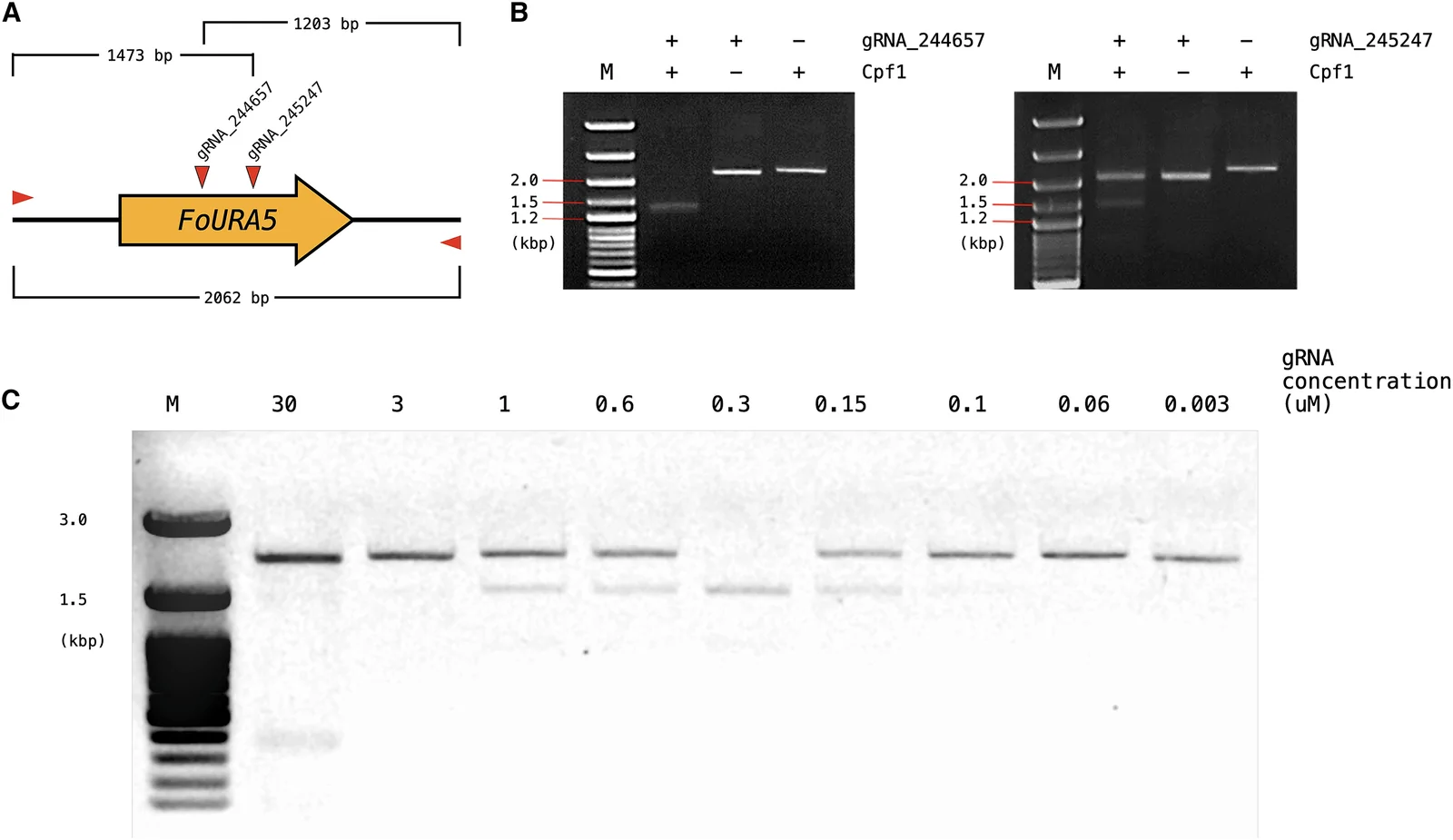
Systematic design and *in vitro* validation of *URA5*-targeting gRNAs for CRISPR/Cpf1 (A) Schematic diagram illustrating the *FoURA5* gene structure, with target sites for gRNA_244657 and gRNA_245247 marked by arrows. The expected cleavage fragments are 1,203 bp for gRNA_244657 and 1,473 bp for gRNA_245247, with the total genomic region spanning 2,062 bp. Primer binding sites are indicated with red triangles. (B) *In vitro* cleavage assays for both gRNAs using purified PCR-amplified DNA fragment. Reactions were performed with (+) and without (−) gRNA or Cpf1 components. M denotes a 1 kbp DNA ladder. (C) Concentration-dependent cleavage analysis demonstrating gRNA activity across serial dilutions from 30 to 0.003 μM. M represents a 100 bp DNA ladder.

The *URA5* gene was selected as the CRISPR/Cpf1 target due to its well-characterized phenotype and utility as a negative selection marker (Figure 3). gRNA (guide RNA) design and validation identified two optimal target sites within the *URA5* coding sequence, with both gRNA_244657 and gRNA_245247 demonstrating robust *in vitro* cleavage activity. Concentration-dependent analysis showed detectable cleavage activity at concentrations between 0.15 and 1 μM, while activity was not clearly observed at higher concentrations (between 3 and 30 μM) or below 0.15 μM.

### GFP-based screening reveals homology arm length-dependent editing efficiency

**Figure 4:**
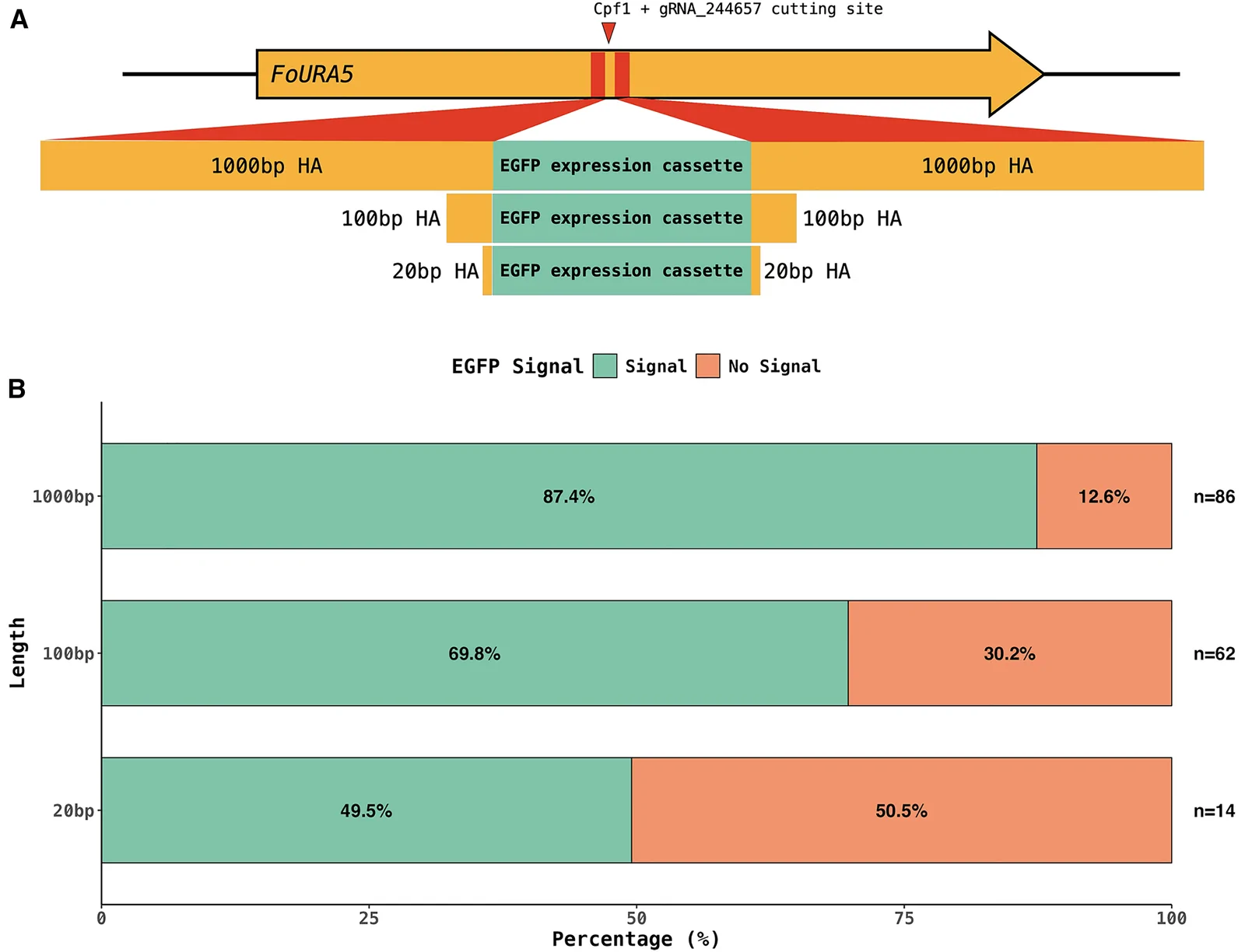
Screening for *ΔURA5* lines with EGFP signals using different homology arm lengths (A) Schematic illustrating the design of an EGFP expression cassette integrated into the *FoURA5* locus via homology-directed repair. The ribonucleoprotein (RNP) complex cuts at the target site within *FoURA5* to facilitate donor integration flanked by homology arms of defined lengths. (B) Proportion of EGFP-positive colonies among 5′-FOA-resistant transformants using donor constructs with different homology arm lengths (20, 100, and 1,000 bp). All colonies were pre-selected on 5′-FOA medium, indicating successful *URA5* gene disruption and uracil auxotroph. Stacked horizontal bars show the percentage of *ΔURA5* colonies with EGFP signal (green, indicating donor integration) and without EGFP signal (orange, indicating *URA5* disruption without donor integration). Sample sizes (*n*) indicated for each condition.

To examine whether varying lengths of homologous arms affect the efficiency of homologous recombination in *F. oxysporum*, we generated a series of EGFP expression cassettes flanked by *URA5* homologous arms ranging from 20 bp to 1 kb (Figure 4A). Initially, 5-fluoroorotic acid (5′-FOA) was used to select for potential *ΔURA5* mutants. Subsequently, these 5′-FOA-resistant transformants were screened using a fluorescence imaging system to detect whether the *EGFP* gene was successfully expressed. Systematic evaluation of homology arm requirements using EGFP reporter integration revealed clear differences in efficiency across arm lengths (Figure 4B). Transformants with 1,000-bp homology arms exhibited 87.4% EGFP-positive colonies, while 100-bp arms achieved 69.8% efficiency. Most significantly, minimal 20-bp homology arms supported 49.5% EGFP integration, demonstrating that extremely short homology sequences retain functional gene-targeting capability.

### Molecular confirmation reveals precise editing event classification

**Figure 5:**
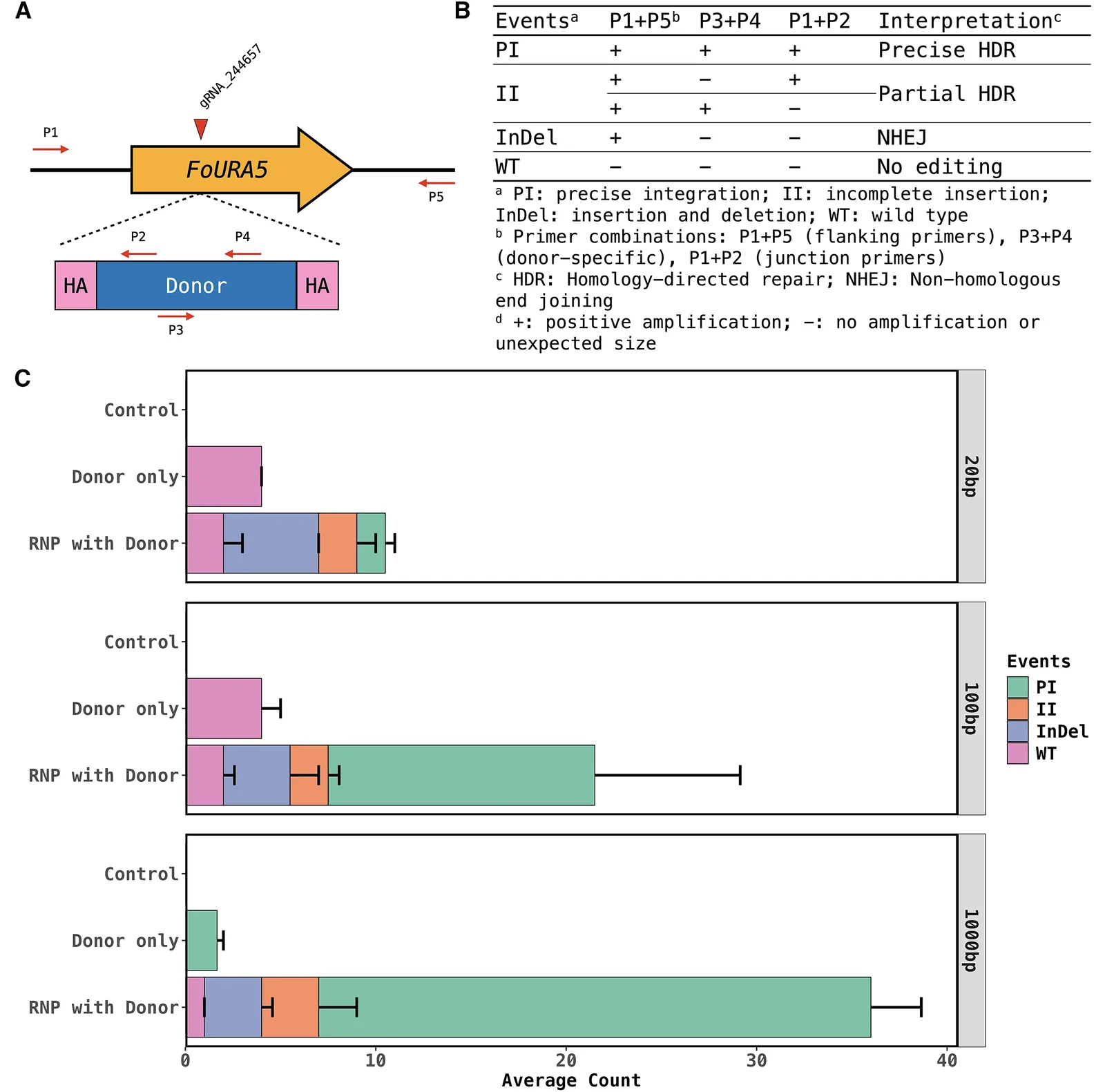
Genotyping for *URA5* editing events (A) Primer design scheme for genotyping analysis. Five primers (P1–P5) are positioned to identify different editing outcomes through PCR amplification patterns. P1+P5 detect gene disruption, P3+P4 identify donor DNA presence, and P1+P2 verify homologous recombination junctions. HA, homologous arms. (B) Classification table for editing events based on PCR results from three primer combinations. SI, precise integration; II, incomplete insertion; InDel, insertion and deletion; WT, wild type. (C) Quantitative analysis of editing outcomes with different homology arm lengths (20, 100, and 1,000 bp). Bars display average counts of each editing event type among transformants under three conditions: control, donor only, and RNP with donor. Color coding corresponds to different mutation types as defined in (B). Data are presented as mean ± SEM from three independent biological replicates.

We next analyzed the editing outcomes in EGFP-expressing transformants. A comprehensive genotyping analysis was performed using a three-primer-pair system to systematically classify editing outcomes (Figure 5). The primer design allowed differentiation between precise integration, partial insertions, and indel formation (Figures 5A and S2). Classification of editing events showed that using 20-bp homology arms resulted in 13.1% of all transformants with precise integrations, while longer homology arms increased the accuracy proportionally (Figures 5B and 5C). A comparative experiment using hygromycin selection with 20-bp homology arms revealed that the majority of hygromycin-resistant (Hyg^R^) colonies resulted from random insertion rather than precise integration (Figure S3). This highlights the importance of the selection method or system when using minimal homology arms.

### Functional validation of EGFP reporter integration

To validate the functionality of EGFP reporter integration and demonstrate its utility for biological applications, EGFP-expressing *URA5-*edited strains were tested in a plant colonization assay. Unlike simple *in vitro* fluorescence detection, this approach validates reporter stability and functionality in the complex environment encountered during plant-microbe interactions.

**Figure 6:**
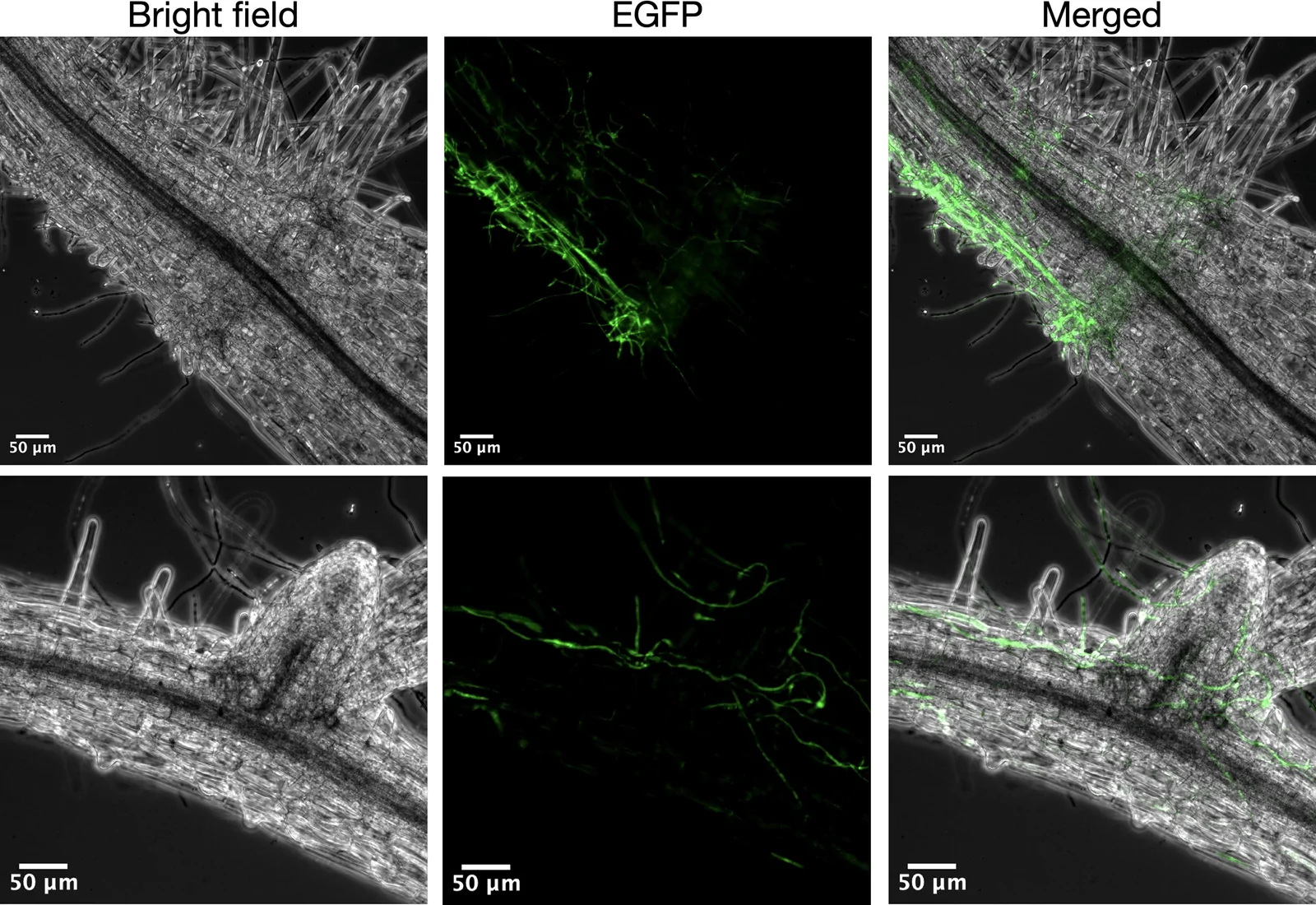
EGFP-tagged *Fusarium oxysporum* enables visualization of plant colonization Fluorescence microscopy of EGFP-expressing *URA5*-edited strains on *Arabidopsis* roots (7 dpi). Images show bright-field (left), EGFP fluorescence (center), and merged channels (right). Top and bottom image representative fields demonstrating stable EGFP expression during root colonization. Fungal structures appear green; plant tissue in grayscale. Scale bars: 50 μm.

Microscopic analysis demonstrated that our editing methods not only achieved the precise gene editing but also maintained stable EGFP expression throughout the colonization process (Figure 6). Control experiments confirmed the expected absence of fluorescence in wild-type strains and uninoculated plants (data not shown).

## Discussion

The small-scale protoplast preparation notably reduces enzyme consumption while maintaining comparable yields, requiring only 7 mg of Driselase compared to 70–800 mg in traditional protocols across various *Fusarium* species (Table 1). This significant reduction in enzyme use, combined with simplified small-scale processing, overcomes a major economic barrier that has limited large-scale functional studies. The faster workflow, enabling edited strains to be obtained within days instead of weeks, results from the integrated rapid monitoring of cell wall regeneration and more efficient transformation protocols. These combined enhancements offer considerable advantages for systematic functional studies, potentially allowing researchers to perform gene function analyses that were previously restricted by resource constraints. The filter column design integrates mycelium confinement, sequential washing, and protoplast recovery within a single device, eliminating multiple transfer steps that are common sources of cell loss and mechanical damage in conventional protocols. Importantly, a single preparation yields sufficient protoplasts to support 2–6 independent transformation experiments. This demonstrates that the method is both economical and practical for routine use.

**Table 1:** Comparison of Driselase usage in fungal protoplast preparation

Study	Organism/strain	Driselase (mg)	Volume (mL)	Yield (×10^8^/mL)	Additional enzymes
This study	*Fusarium oxysporum* f. sp. *conglutinans*	7	0.7	0.3–0.8	β-glucanase
Yang et al.^16^	*F. oxysporum* f. sp. *fragariae* (H6)	70–100	10	not reported	cellulase, lysozyme
Shi et al.^17^	*F. oxysporum* (FO-1)	500	25	1.44	none
Ayukawa et al.^18^	*F*. *oxysporum* f. sp. *conglutinans*	500	25	4	lysing enzymes
Shao et al.^19^	*F. graminearum* (PH-1)	100	20	not reported	lysing enzymes, snailase
Hicks et al.^20^	*F. graminearum* (DAOMC233423)	500	20	2	lysing enzymes, yatalase
Lohmar et al.^21^	*F. verticillioides* (Fv999)	800	20	5–10	lysing enzymes, chitinase

Our demonstration that 20-bp homology arms support functional gene targeting represents a significant advancement over conventional CRISPR approaches, which typically require 500–2,000 bp homology arms for reliable HDR (homolog directed repair) in fungi.^11^ This capability stems from key biochemical differences between Cpf1 and Cas9 systems. Most significantly, Cpf1 generates staggered DNA breaks with 4–5 nucleotide overhangs that facilitate homologous recombination, whereas Cas9 produces blunt-end cuts that can activate error-prone NHEJ pathways.^22^ Additionally, Cpf1 utilizes shorter guide RNAs that require no complex scaffold structure, substantially simplifying the design and synthesis of targeting constructs compared with the Cas9-based system.^23^

The 13.1% precise HDR efficiency achieved through our *URA5*/5′-FOA selection strategy demonstrates that these advantages translate into practical benefits, with the two-step selection approach effectively enriching for editing events. The comparison of selection strategies revealed that combining minimal-homology arms with antibiotic selection substantially increases the frequency of random genomic integration. *F. oxysporum* is known to favor NHEJ over homology-directed repair, making random integration an inherent characteristic of this organism.^24^ Under these circumstances, the use of 20-bp homology arms further reduces the competitive advantage of HDR over NHEJ, increasing the likelihood of random insertion events. When combined with Hyg^R^ selection, the strong survival pressure imposed by hygromycin enriches all transformants carrying any genomic integration event, regardless of insertion location. This was confirmed by genotyping of Hyg^R^-resistant colonies (Figure S3). Unlike the successful Hyg^R^-based editing previously reported in *F. oxysporum*,^7^ which relied on substantially longer homology arms, our approach deliberately employs 20-bp homology arms, which require a more stringent counter-selection system to effectively enrich for precise integration events. In contrast, the *URA5*/5′-FOA selection system specifically eliminates colonies that retain functional URA5, regardless of insertion site, thereby ensuring that only precisely edited events are recovered. These findings demonstrate that, under minimal-homology-arm conditions, a counter-selection strategy is essential to overcome random integration. Conventional preparation of lengthy homology arms requires several weeks of cloning, whereas a 20-bp sequence can be incorporated via oligonucleotide synthesis within hours, thereby significantly accelerating experimental timelines.

The platform’s utility extends beyond fundamental research to support agricultural biotechnology objectives. The generation of an EGFP-marked strain demonstrates real-time monitoring capabilities for host-pathogen interaction studies.^25^ This enables dynamic visualization of colonization processes that advance traditional plant pathology methodologies. The established *Arabidopsis* inoculation system provides a standardized phenotypic validation framework, facilitating the assessment of gene function and spatial expression patterns during pathogenesis.^26^ The combination of rapid mutant generation, cost-effective screening, and reliable validation creates a framework for systematic knockout studies that provides a foundation for identifying virulence factors and potential biocontrol targets, supporting biotechnology development tailored to agricultural challenges.

### Limitations of the study

Several constraints limit the broader applicability of this platform. Protoplast-based transformation remains technically demanding due to cell fragility and regeneration variability, impacting reproducibility particularly in high-throughput applications. The 13.1% precise HDR efficiency with 20-bp arms, while functional, may limit its use in applications requiring high-frequency modification. A significant limitation is the dependence on the selection system design: favorable results with URA5 benefit from 5′-FOA-negative selection, but targeting other genes would rely primarily on EGFP screening, potentially requiring screening of significantly more transformants to identify precise editing events. Current validation, limited to URA5 in *F. oxysporum*, necessitates a comprehensive evaluation across diverse targets and fungal species to establish general utility. For targeting genes other than *URA5*, alternative approaches could be considered, including the use of *URA5-*disrupted strains as target strains by knockin expression of *URA5* in the target region.

## Resource availability

### Lead contact

Requests for further information and resources should be directed to and will be fulfilled by the lead contact, Tao-Ho Chang (thchang@nchu.edu.tw).

### Materials availability

All unique/stable reagents generated in this study are available from the lead contact without restriction.

### Data and code availability

• All data reported in this paper will be shared by the lead contact upon request.
• This paper does not report original code.
• Any additional information required to reanalyze the data reported in this paper is available from the lead contact upon request.

## Acknowledgments

This work was financially supported by the 10.13039/100020595National Science and Technology Council, Taiwan, under grant number 112-2313-B-005-008-, 114-2313-B-005-018-MY3, and 114-2637-B-020-002-. We extend sincere appreciation to colleagues at the Academy of Circular Economy, National Chung Hsing University, for their invaluable guidance and technical assistance throughout this research. Special thanks are given to Prof. Jenn-Wen Huang and Prof. Pi-Fang Linda Chang for their essential contributions during the initial stages of this study, including the provision of *F*. *oxysporum* strains and critical research materials that enabled this investigation. Additional acknowledgment is given to the technical staff and laboratory personnel who provided experimental support and assistance with laboratory procedures.

## Author contributions

Conceptualization, J.-Z.Z. and T.-H.C.; methodology, J.-Z.Z. and T.-H.C.; investigation, J.-Z.Z., S.-C.H., and W.-T.Z.; writing – original draft, J.-Z.Z.; writing – review and editing, Y.-H.L. and T.-H.C.; funding acquisition, Y.-H.L. and T.-H.C.; resources, Y.-H.L. and T.-H.C.; supervision, T.-H.C.

## Declaration of interests

The authors declare no competing interests.

## Declaration of generative AI and AI-assisted technologies in the writing process

During the preparation of this work, the authors used Claude (sonnet 4.5) to improve readability. After using this tool or service, the authors reviewed and edited the content as needed and take full responsibility for the content of the publication.

## STAR★Methods

### Key resources table

**Table:** 

REAGENT or RESOURCE	SOURCE	IDENTIFIER
**Bacterial and virus strains**		

*Escherichia coli* DH5α	Yeastern Biotech Co., Ltd	Cat#FYE607-80VL

**Biological samples**		

*Fusarium oxysporum* f.sp. *conglutinans*	Dr. Jenn-Wen Huang, National Chung Hsing University	FCN33

**Chemicals, peptides, and recombinant proteins**		

Sucrose	BIO BASIC	Cat#SB0498
Yeast extract	Laboratorios Conda S.A	Cat#1702.00
Hy-Case® SF	Sigma-Aldrich	Cat#C9386
Agar	Cyrusbioscience	Cat#101-9002-18-0
PEG (polyethylene glycol) 3350	HAMPTON RESEARCH	Cat#HR2-591
NaCl	BioShop	Cat#SOD002.1
Driselase™ from *Basidiomycetes* sp.	Sigma-Aldrich	Cat#D9515-5G
β-Glucanase from *Trichoderma longibirachiatum*	Sigma-Aldrich	Cat#G4423-100G
MgSO_4_.7H_2_O	BIO BASIC	Cat#MB0329
KCl	EMPEROR CHEMICAL	Cat#P190612006442
FeSO_4_.7H_2_O	EMPEROR CHEMICAL	Cat#20130301050
NaNO_3_	UNION CHEMICAL	Cat#85623
Citric acid, Monohydrate	GeneMark	Cat#GJH14138-030
ZnSO_4_.7H_2_O	EMPEROR CHEMICAL	Cat#20130301025
Fe(NH_4_)_2_(SO_4_).12H_2_O	Scharlau	Cat#HI03150500
CuSO_4_.5H_2_O	EMPEROR CHEMICAL	Cat#20130301031
MnSO_4_.H_2_O	EMPEROR CHEMICAL	Cat#20130301038
H_3_BO_4_	EMPEROR CHEMICAL	Cat#20130208016
NaMoO_4_.2H_2_O	EMPEROR CHEMICAL	Cat#20130304074
Uridine	BIO BASIC	Cat#UD0570
Uracil	BIO BASIC	Cat#UD0564
5-Fluoroorotic acid	Cyrusbioscience	Cat#101-703-95-7
Cetyltrimethylammonium bromide	Alfa Aesar	Cat#A15235.22
Murashige & Skoog Basal Medium with Vitamins (MS)	PhytoTech Labs	Cat#M519
Potato dextrose broth	HiMedia	Cat#GM096
Agarose	Cyrusbioscience	Cat#101-9012-36-6
Gellan gum	Cyrusbioscience	Cat#3012
Tris buffer (1.5M, pH = 8.8)	Biomate	Cat#BR270

**Critical commercial assays**		

Blend Taq™ High Efficient Taq DNA polymerase	TOYOBO	Cat#BTQ-101
EnGenⓇ Lba Cas12 a (Cpf1)	New England Biolabs	Cat#M0653S
FavorPrep™ Plasmid Extraction Kit	Favorgen	Cat#FAPDE300

**Experimental models: Organisms/strains**		

*Arabidopsis thaliana* Col-0	Dr. Tao-Ho Chang, National Chung Hsing University, Taiwan	Col-0

**Oligonucleotides**		

See Table S1	This study	N/A

**Recombinant DNA**		

pCR^TM^ 2.1	ThermoFisher	RRID:Addgene_208363
pAN7.1	Addgene	RRID:Addgene_168129
pBI-MCS-EGFP	Addgene	RRID: Addgene_16542
pAN7.1_EGFP	This study	N/A
pAN7.1_*URA5*_donor	This study	N/A

**Software and algorithms**		

RStudio	Posit Software	https://posit.co/downloads/
FIJI	ImageJ	https://imagej.net/software/fiji/
Benchling	Benchling	https://www.benchling.com/

### Experimental model and study participant details

#### Fungal strains

*Fusarium oxysporum* f. sp. *conglutinans* FCN33 (crucifer-adapted) was used in this study. FCN33 was obtained from Dr. Jenn-Wen Huang (National Chung Hsing University, Taiwan). Single spore isolates were obtained using sterile glass needles under a dissecting microscope to ensure genetic homogeneity and culture purity. Stock cultures were preserved in sterile sand tubes containing 2% water agar and approximately 1% sandy soil, stored at room temperature for long-term maintenance. For routine culturing, strains were grown on half-strength potato dextrose agar (1/2 PDA: 19.5 g PDA per liter) at 28°C.

#### Plant material

*Arabidopsis thaliana* Col-0 seeds were surface-sterilized (incubated with 2% sodium hypochlorite for 10 min) for the plate assay. Seeds were stratified at 4°C for 24 h before sowing on half-strength Murashige and Skoog (MS) medium containing 1% agar and 1% sucrose. Plants were grown in a controlled environment chamber at 25°C under 16-h photoperiod.

#### Culture media

M100 selective medium (3% w/v sucrose, 0.5 g/L MgSO_4_.7H_2_O, 0.5 g/L KCl, 10 mg/L FeSO_4_.7H_2_O, 2 g/L NaNO_3_, 2 mL/L trace elements, 2% w/v agar) supplemented with appropriate selection agents. For URA5 selection, 2 mg/mL 5′-fluoroorotic acid (5′-FOA) was added along with 250 μM uridine and uracil. SuTC buffer (20% w/v sucrose, 50 mM Tris-HCl pH 8.0, 50 mM CaCl_2_·H_2_O) was used for protoplast manipulation and storage.

### Method details

#### Small-scale protoplast preparation

Mycelium preparation for protoplast isolation was initiated by inoculating 10^7^ conidia in 50 mL half-strength PDB and incubating at 28°C with 125 rpm shaking for 16–20 h to obtain young mycelium. The culture was filtered through a spin filter column (FavorPrep Genomic DNA Extraction Mini Kit, FAVORGEN) at 1,000×*g* for 30 s to remove growth medium and residual conidia, followed by washing with 700 μL sterile distilled water.

Enzymatic digestion buffer preparation involved testing four different osmotic stabilizing solutions: (A) 0.8 M NaCl, (B) 0.8 M KCl, (C) 10 mM Na_2_HPO_4_, 20 mM CaCl_2_, 1.2 M NaCl, pH 5.8, and (D) 0.6 M mannitol, 10 mM Tris-HCl, 10 mM CaCl_2_, pH 7.5. Each digestion buffer contained 10 mg Driselase (Sigma-Aldrich D9515) and 15 mg β-glucanase (Sigma-Aldrich G4423) per 1 mL total volume. All digestion buffers were filter-sterilized through 0.22 μm disc filters before use.

Protoplasts were generated by adding 700 μL of digestion buffer to the washed mycelium in the filter column. The mixture was gently pipetted until mycelium was fully suspended, then the column was inverted and incubated at 28°C with 125 rpm rotary shaking for 2 h. Following digestion, the column was centrifuged at 5,000 × g for 10 min at 4°C to collect protoplasts in the collection tube. The filter column was carefully removed and the protoplast pellet was washed twice with 1 mL SuTC buffer by gentle centrifugation at 5,000×*g* for 5 min at 4°C. Protoplasts were resuspended in 500 μL SuTC buffer and counted using a hemocytometer, then diluted to 10^7^ protoplasts per mL for transformation experiments.

Cell wall regeneration monitoring was performed using calcofluor white staining at 0, 1, 2, 3, and 4 h post-isolation to assess cell wall reconstruction kinetics and determine optimal transformation timing.

#### Fungal DNA extraction and target gene cloning

Approximately 100 mg of fresh mycelium from solid medium was frozen in liquid nitrogen and then homogenized using a bead mill homogenizer (Precellys 24 Touch) at 6800 rpm for 40 s. The DNA extraction procedure followed the instructions from the FavorPrep Plant Genomic DNA Extraction Kit (FAVORGEN). Quality and quantity of DNA were measured using the ratio of wavelength 260/230 and 260/280 by Nanodrop (Biometrics).

The polymerase chain reaction (PCR) was performed with 2X Taq DNA Polymerase Master Mix (AMPLIQON), and the reaction setup was according to the instructions. The PCR products were loaded in 1% agarose gel containing 0.01% safety staining dye (SYBR Safe DNA Gel Stain, Invitrogen) for running gel electrophoresis. The ideal size of bands was purified by gel purification kit (Favorgen), and the quality of purified PCR products were examined by Nanodrop.

For *FoURA5* cloning, 1 μL of purified PCR product was mixed with TOPO TA Cloning Vector (Thermo Fisher) according to the manual script. After incubating at room temperature for 5 min, 2 μL reaction was added in 50 μL DH5ɑ competent cells (ECOS), and the following procedure is based on the standard heat-shock transformation protocol. A primer pair for amplifying the coding region of *FoURA5* was listed in Table S1.

#### gRNA design *and in vitro* validation

Target site selection was performed using the *FoURA5* gene sequence as the template for CRISPR/Cpf1 targeting. The *URA5* gene encodes orotate phosphoribosyltransferase, essential for pyrimidine biosynthesis, and serves as an effective negative selection marker when disrupted. Potential guide RNA target sites were identified using Benchling CRISPR design platform (Benchling Inc., San Francisco, CA), with selection criteria including optimal TTTV protospacer adjacent motif (PAM) positioning and minimal predicted off-target effects within the *F. oxysporum* genome.

Two guide RNAs were designed: gRNA_244657 and gRNA_245247, targeting distinct sites within the *FoURA5* coding sequence. Guide RNAs were synthesized as 23 nucleotide (nt) sequences with 20 nt additional scaffold specific for Cpf1. Synthetic guide RNAs were obtained from MDBio (Taichung, Taiwan) and dissolved in nuclease-free water to 100 μM stock concentrations.

*In vitro* cleavage assays were performed to validate gRNA activity before transformation experiments. Genomic DNA template was prepared by PCR amplification of the *FoURA5* target region using primers P1 and P5, generating a 2,062 bp fragment containing both gRNA target sites. Cleavage reactions contained 500 ng template DNA, varying concentrations of gRNA (0.003–30 μM), and 5 μg LbCas12a protein (EnGen Lba Cas12a, New England Biolabs M0653S) in 1× reaction buffer. Reactions were incubated at 37°C for 1 h, then analyzed by agarose gel electrophoresis to assess cleavage efficiency. Expected cleavage products were 1,203 bp for gRNA_244657 and 1,473 bp for gRNA_245247.

Concentration optimization experiments demonstrated complete target cleavage at gRNA concentrations between 1 and 3 μM, with detectable activity maintained at concentrations as low as 0.15 μM. Based on these results, gRNA_244657 showed superior cleavage efficiency and was selected for subsequent transformation experiments.

#### CRISPR/Cpf1 ribonucleoprotein complex assembly

Ribonucleoprotein (RNP) complex preparation was performed immediately prior to protoplast transformation to ensure optimal activity. Pre-assembly reactions contained 500 ng of selected guide RNA (gRNA_244657), 5 μg LbCas12a protein (EnGen Lba Cas12a, New England Biolabs M0653T), and 1× reaction buffer in a total volume of 20 μL. The mixture was incubated at 25°C for 15 min to allow RNP complex formation, following established protocols for ribonucleoprotein assembly.

Donor DNA construct preparation involved generating EGFP reporter cassettes with varying homology arm lengths to systematically evaluate HDR efficiency. The EGFP expression cassette was amplified from pAN7.1-EGFP vector and flanked with *FoURA5* homology sequences of 20 bp, 100 bp, and 1,000 bp at both 5′ and 3' ends. For 20 bp homology arms, sequences were directly incorporated into PCR primers during amplification. For longer homology arms, sequential PCR reactions were performed using nested primer strategies to extend homologous sequences progressively.

PCR amplifications were performed using KAPA HiFi DNA Polymerase (KAPA Biosystems) according to manufacturer’s instructions, with products purified using PCR purification kits (FavorPrep, FAVORGEN). All donor DNA constructs were verified by agarose gel electrophoresis and quantified using spectrophotometric analysis before use in transformation experiments. Typical donor DNA concentrations of 200–500 ng/μL were used, with 2 μg total donor DNA per transformation reaction.

Transformation mixture preparation involved combining pre-assembled RNP complexes with 200 μL protoplast suspension (10^7^ cells/mL) and 2 μg donor DNA in a 50 mL tube. The mixture was incubated at room temperature for 20 min to allow RNP-protoplast interaction before PEG-mediated transformation.

#### Protoplast transformation and selection

PEG-mediated transformation was performed using a modified protocol optimized for *F. oxysporum* protoplasts. Following RNP-protoplast incubation, 1 mL of PTC solution (60% polyethylene glycol 4000 in SuTC buffer) was slowly added dropwise to the transformation mixture while gently flicking the tube bottom to ensure gradual mixing. The transformation reaction was incubated at room temperature for 20 min to facilitate membrane permeabilization and DNA uptake.

Cell recovery and regeneration involved diluting the transformation mixture with 5 mL liquid TB3 medium and incubating at 28°C for 12-18 h to allow cell wall regeneration and metabolic recovery. This recovery period is critical for protoplast viability and subsequent selection marker expression.

Selection procedures utilized the *URA5* disruption phenotype for identifying successful transformants. M100 selective medium was prepared by melting 45 mL M100 base medium to 55°C and adding 5-fluoroorotic acid (5′-FOA) to 2 mg/mL final concentration, along with 250 μM uridine and uracil as metabolic supplements. The recovered transformation mixture was gently mixed with the selective medium and poured into sterile Petri dishes before solidification. Plates were incubated at 28°C for 10-14 days to allow colony development.

EGFP-based primary screening enabled rapid identification of transformants containing integrated donor DNA (Figure S4). Colonies were examined under blue light excitation using a fluorescence image system (iBright 1500, Invitrogen) to identify EGFP-positive clones. The percentage of fluorescent colonies among total 5′-FOA resistant transformants was calculated for each homology arm length condition. Representative EGFP-positive and EGFP-negative colonies were selected for molecular confirmation and preserved as glycerol stocks at −80°C.

Transformation efficiency was calculated as the number of 5′-FOA resistant colonies per 10^6^ protoplasts transformed, with EGFP integration efficiency determined as the percentage of fluorescent colonies among total resistant transformants.

#### Molecular genotyping and confirmation

Genomic DNA extraction from selected transformants was performed using the FavorPrep Genomic DNA Extraction Mini Kit (FAVORGEN) according to manufacturer’s instructions. Fungal mycelia were collected from 3-day-old liquid TB3 cultures and processed immediately to ensure high-quality DNA recovery.

PCR-based genotyping strategy utilized a comprehensive three-primer-pair system designed to distinguish different editing outcomes at the *FoURA5* locus. Five primers were strategically positioned around the target site: P1 and P5 flanked the entire *FoURA5* gene region, P3 and P4 were located within the EGFP donor sequence, and P1 and P2 spanned the 5′ homologous recombination junction. This primer design enabled systematic classification of editing events through differential PCR amplification patterns.

Mutation event classification was performed using three primer combinations with distinct diagnostic capabilities: P1+P5 amplification confirmed gene disruption events, P3+P4 amplification detected donor DNA integration, and P1+P2 amplification verified precise homologous recombination at the target junction. PCR reactions were performed using Blend Taq DNA Polymerase with the following cycling conditions: 95°C for 5 min initial denaturation, followed by 35 cycles of 95°C for 30 s, 58°C for 30 s, and 72°C for 2 min, with a final extension at 72°C for 10 min.

Editing outcome categories were defined based on PCR amplification patterns: (1) Precise integration (SI): successful amplification with all three primer pairs indicating precise HDR, (2) Incomplete Insertion (II): P1+P5 and P3+P4 positive but P1+P2 negative, suggesting partial donor integration, (3) InDel events: P1+P5 positive but P3+P4 and P1+P2 negative, indicating NHEJ-mediated mutations, and (4) Wild Type (WT): amplification patterns consistent with unedited sequence.

Molecular confirmation involved analyzing at least 10–15 randomly selected transformants from each experimental condition. PCR products were visualized by agarose gel electrophoresis using 1% agarose gels stained with ethidium bromide, with molecular weight markers included for size verification. Representative PCR products were purified and subjected to Sanger sequencing to confirm sequence accuracy and verify precise integration events.

#### Plant colonization and fluorescence imaging

*Arabidopsis thaliana* seeds (ecotype Col-0) were sterilized in 10% bleach for 10 min, rinsed 10 times with sterile water, and vernalized at 4°C in darkness overnight. The seeds were germinated and grown on half-strength MS medium (½MS, 2.16 g L^−1^ MS salts, 1% Gellan gum, 1% sucrose) at 22°C–25°C under a 16-h light cycle for 7 days. Seven-day-old plants were then transferred to ½ MS without sucrose and subjected to Fusarium inoculation.

Fungal inoculum was prepared by harvesting conidia from 7-day-old cultures grown on half-strength PDA plates. Conidia were collected by flooding plates with sterile distilled water and gently scraping the surface with a sterile spatula. The conidial suspension was filtered through four layers of Miracloth and adjusted to 10^6^ conidia/mL using a hemocytometer.

The inoculation protocol involved adding 100 μL of a conidial suspension (10^5^ conidia final concentration) directly to the root zone of each seedling. Control treatments received 100 μL of sterile distilled water. Inoculated plants were maintained in the controlled environment chamber at 22°C under 16-h photoperiod conditions for 7 days post-inoculation.

Microscopic colonization analysis was performed on EGFP-expressing strains to visualize fungal invasion and tissue colonization patterns. At 7 days post-inoculation, infected root segments were excised and examined using fluorescence microscopy with appropriate filter sets for EGFP detection. Representative images were captured at different root locations including the collet region, intercellular spaces, lateral root junctions, and vascular tissues to document colonization diversity. Images were acquired using a fluorescence microscope (Axiovert 5 with Axiocam 705 mono detector; ZEISS, Germany). For EGFP visualization, the following optical configuration was used: excitation filter 470/40 nm (470 nm center wavelength, bandwidth 40 nm), emission filter 525/50 nm (525 nm center wavelength, bandwidth 50 nm). Images were captured using automatic exposure setting optimized for each channel independently. Both bright-field and fluorescence images were acquired from the same field of view. The fluorescence images were false-colored green to represent the EGFP signals.

### Quantification and statistical analysis

Sample sizes and experimental replication varied by experimental objective, with all experiments performed in biological triplicates unless otherwise specified. In protoplast optimization experiments, n represents individual protoplast preparation reactions, with at least 6 independent preparations per condition. All experiments in this manuscript were repeated at least three times. Each experiment has at least 3 replicate biological samples.

Data are presented as descriptive statistics (mean ± standard error of the mean, SEM). Individual data points are displayed alongside summary statistics to demonstrate biological variability. Error bar represent SEM unless otherwise specified. Data analysis, including computation of descriptive statistic (mean, SEM) and data visualization, was performed using in RStudio with R statistical software (version 4.3.0) with appropriate packages.^27^ Graphs were generated using ggplot2 package for clear presentation of results.

No statistical significance testing was performed. No data points were excluded from analyses except where technical failures (e.g., contamination, equipment malfunction) were explicitly documented during experimental procedures.
