# Supplementary material for: A streamlined protocol for small-scale protoplast generation and CRISPR/Cpf1-mediated genome editing in Fusarium oxysporum
Source: iScience. 2026 Aug 27;29(9):117365. doi: 10.1016/j.isci.2026.117365 (PMC13546873; doi:10.1016/j.isci.2026.117365)
Supplement: Supplementary file 1 [file mmc1.pdf]

iScience, Volume *29*

## Supplemental information

# A streamlined protocol for small-scale protoplast generation and CRISPR/Cpf1-mediated genome editing in *Fusarium oxysporum*

**Jun-Ze Zheng, Szu-Chieh Huang, Wen-Ting Zeng, Ying-Hong Lin, and Tao-Ho Chang**

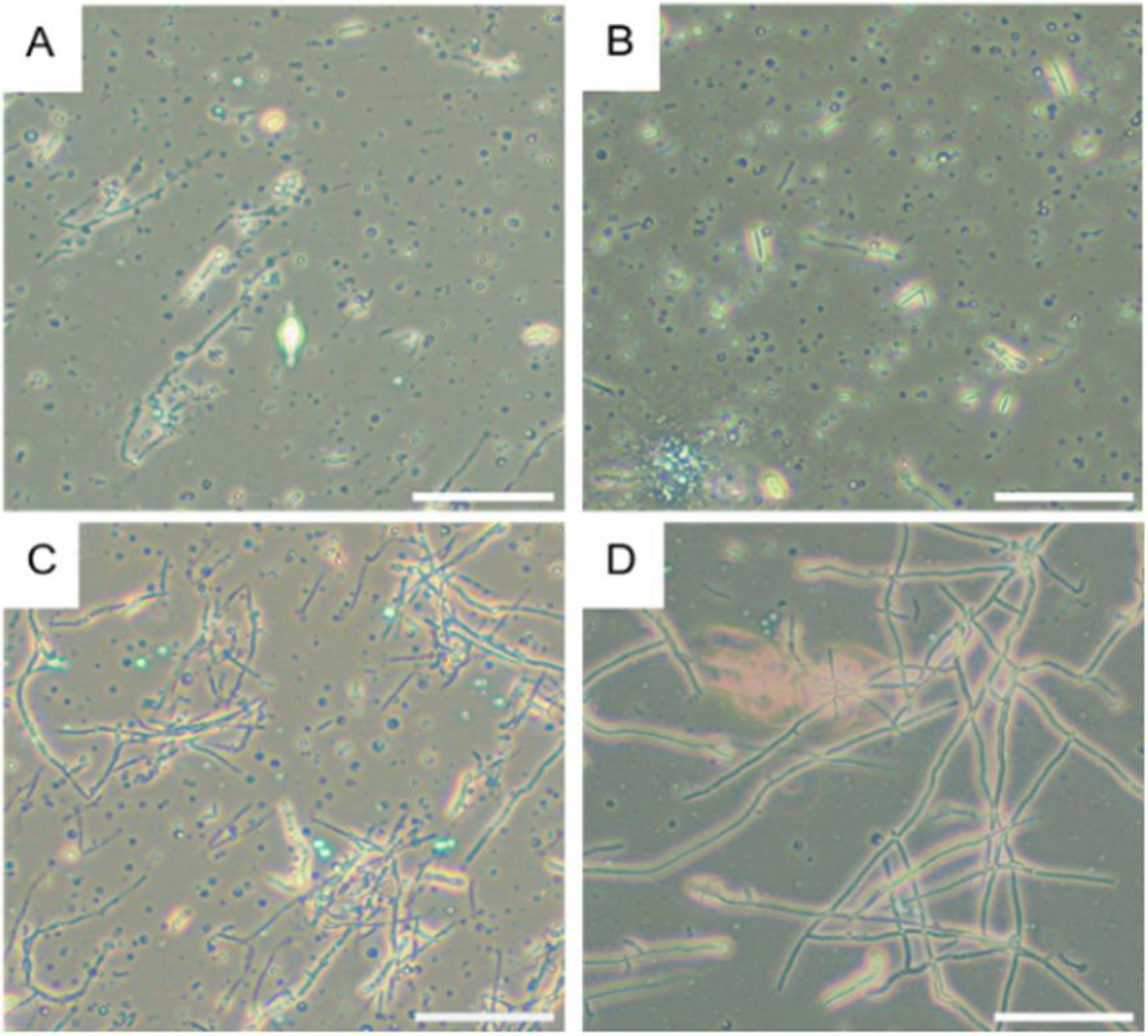


**Figure S1. Microscopic results of protoplasts in different stabiliser**
Different stabiliser (A: 0.8 M NaCl; B: 0.8 M KCl; C: 10 mM $Na_2PO_4$, 20 mM $CaCl_2$, 1.2 M NaCl, pH 5.8; D: 0.6 M mannitol, 10 mM $CaCl_2$, 10 mM Tris-HCl) were applied for examining the small scale protoplast generation. Scale bars at bottom left corner represent 20 μm.

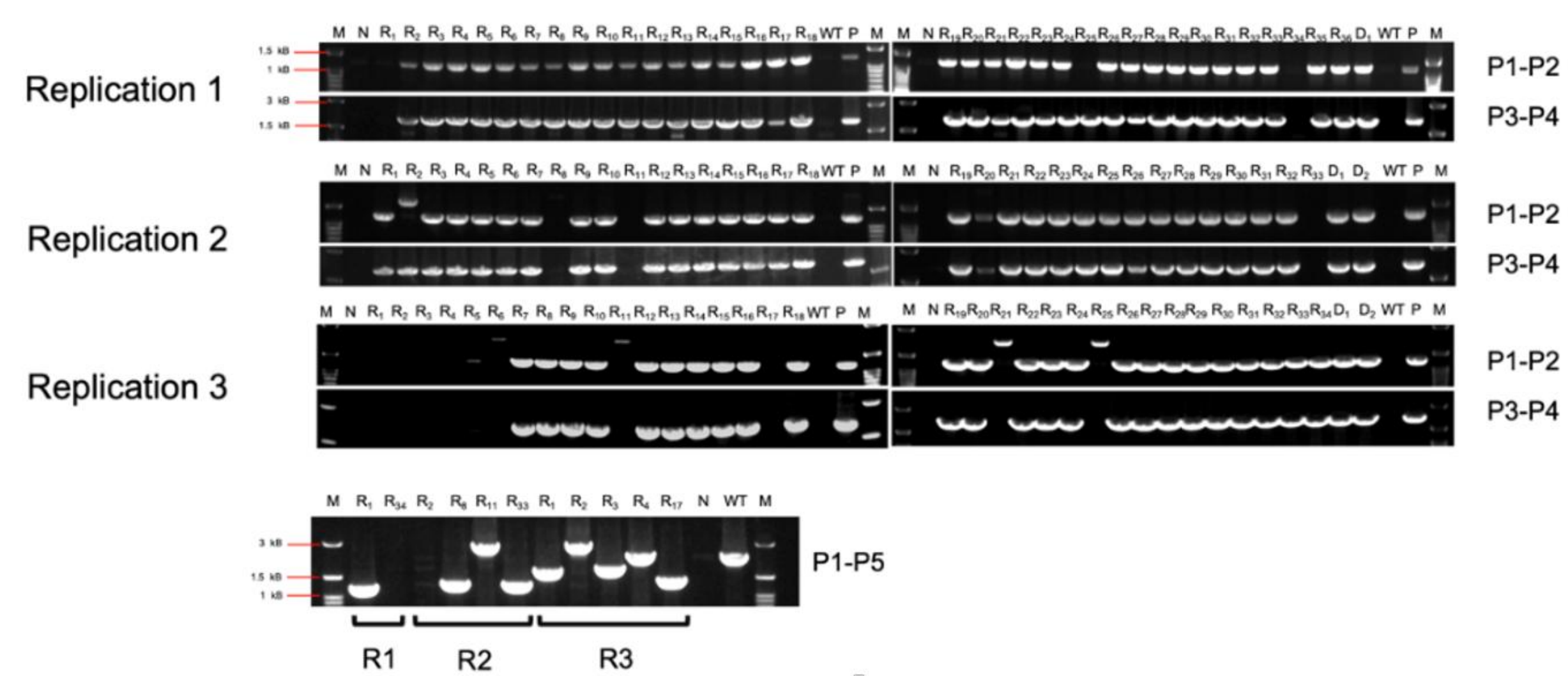


**Figure S2. Gel electrophoresis for genotyping**
Electrophoresis gel examined the polymerase chain reaction (PCR) to determine the genotyping of editing strains.

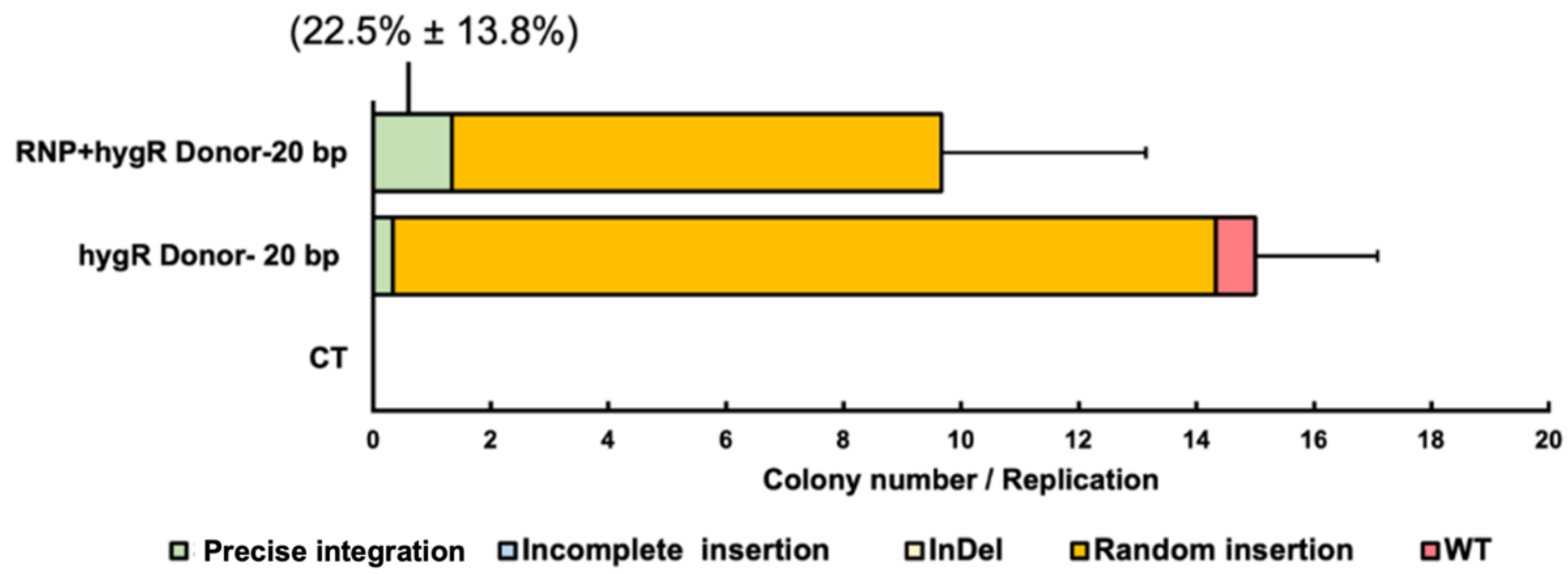


**Figure S3. Genotyping of 20 bp homologous arms for integration the $Hyg^R$ expression donor**

The 20 bp-length homologous arms integration donor with hygromycin resistance gene, showed the variant genotypes of the edited strains. Colour coding corresponds to different mutation types as defined in bottom panel. Data are presented as mean ± SEM from three independent biological replicates.

| Bright field | Fluorescent | Merged |
|---|---|---|
| 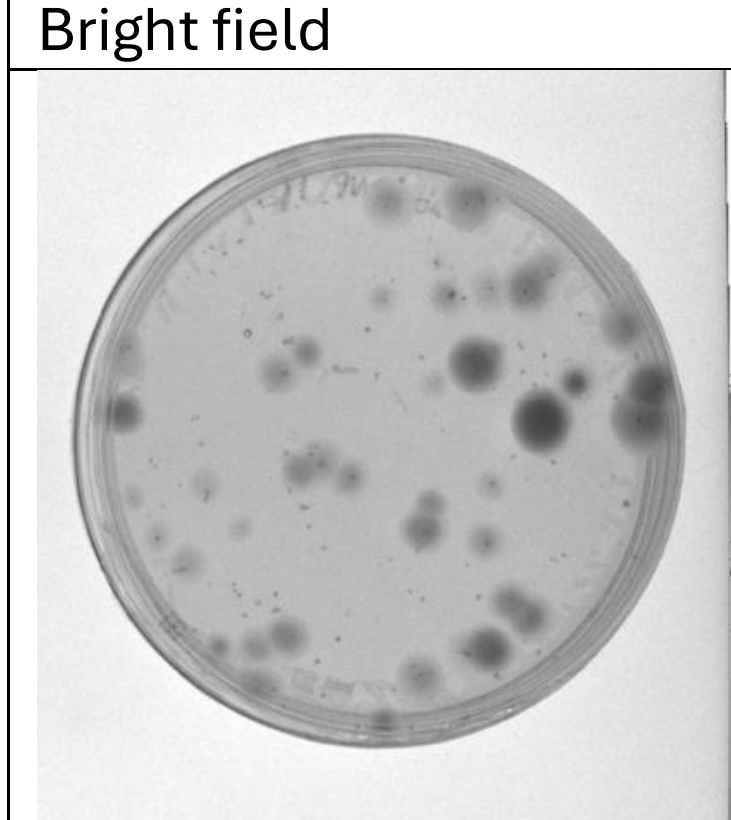 | 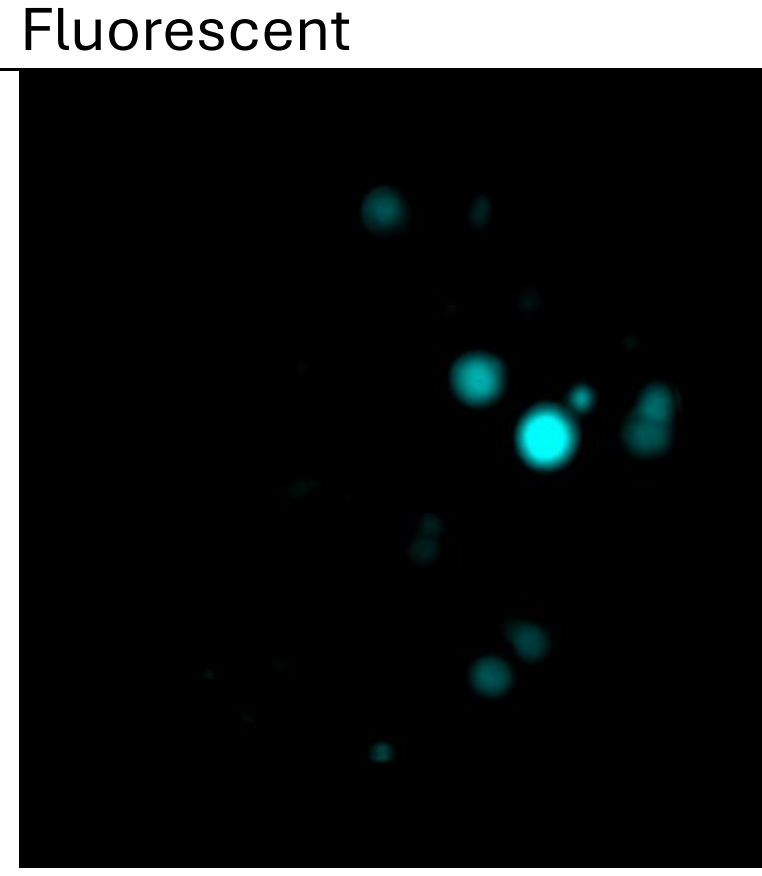 | 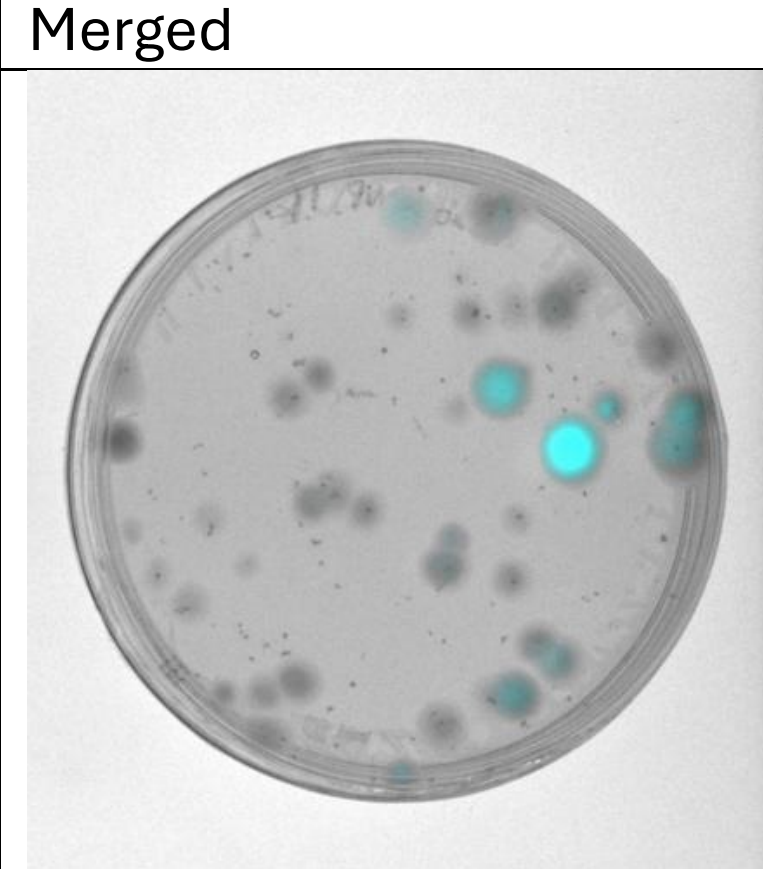 |

**Figure S4. Using fluorescent image to select potential mutants**

The 5'-FOA-resistant transformants were examined using the iBright fluorescence imaging system. EGFP-positive transformants were isolated for further analysis. The plate size is 9 cm diameter.

**Table S1. Oligonucleotides used in this study**

| Name | Sequence | Source |
|---|---|---|
| gRNA_244657 | TAATTTCTACTAAGTGTAGATGAAATATTCACTCC CAATCTTGA | This study |
| gRNA_245247 | TAATTTCTACTAAGTGTAGATGCAACTCGCCAATG GCACTGGGG | This study |
| FoURA5_F | AAATGGTCGGCATCGTAGAG | This study |
| FoURA5_R | TCAAAGACCTTGGCCCAAGC | This study |
| P1 | AAATGGTCGGCATCGTAGAG | This study |
| P2 | GCTTGTGTTGTGTGACTTTTGG | This study |
| P3 | AAATATCGTGCCTCTCCTGC | This study |
| P4 | TTACTTGTACAGCTCGTCCATG | This study |
| P5 | TCAAAGACCTTGGCCCAAGC | This study |
